# Development of a Targeted Flip-in System in Avian DT40 Cells

**DOI:** 10.1371/journal.pone.0122006

**Published:** 2015-03-23

**Authors:** Kaori Kobayashi, Toshihiko Fujii, Ryuta Asada, Masato Ooka, Kouji Hirota

**Affiliations:** Department of Chemistry, Graduate School of Science and Engineering, Tokyo Metropolitan University, Minamiosawa 1-1, Hachioji-shi, Tokyo 192-0397, Japan; Chang Gung University, TAIWAN

## Abstract

Gene-targeting to create null mutants or designed-point mutants is a powerful tool for the molecular dissection of complex phenotypes involving DNA repair, signal transduction, and metabolism. Because gene-targeting is critically impaired in mutants exhibiting attenuated homologous recombination (HR), it is believed that gene-targeting is mediated via homologous recombination, though the precise mechanism remains unknown. We explored gene-targeting in yeast and avian DT40 cells. In animal cells, gene-targeting is activated by DNA double strand breaks introduced into the genomic region where gene-targeting occurs. This is evidenced by the fact that introducing double strand breaks at targeted genome sequences via artificial endonucleases such as TALEN and CRISPR facilitates gene-targeting. We found that in fission yeast, *Schizosaccharomyces pombe*, gene-targeting was initiated from double strand breaks on both edges of the homologous arms in the targeting construct. Strikingly, we also found efficient gene-targeting initiated on the edges of homologous arms in avian DT40 cells, a unique animal cell line in which efficient gene-targeting has been demonstrated. It may be that yeast and DT40 cells share some mechanism in which unknown factors detect and recombine broken DNA ends at homologous arms accompanied by crossover. We found efficient targeted integration of gapped plasmids accompanied by crossover in the DT40 cells. To take advantage of this finding, we developed a targeted flip-in system for avian DT40 cells. This flip-in system enables the rapid generation of cells expressing tag-fused proteins and the stable expression of transgenes from *OVA* loci.

## Introduction

Deletion and mutation studies are central to the functional analysis of genes in yeast and mice. Such genetic modifications are mediated by gene-targeting. Gene-targeting constructs are introduced into the host chromosome either by targeted or random DNA integration. In yeast, targeted integration dominates, while in mouse embryonic stem (ES) cells, the ratio of targeted to random integration is 1:300 to 40000 [1–11]. Most transfected mouse ES cells are thus not targeted, and the generation of mutant cells in mice is significantly more difficult than in yeast. Because the avian DT40 B cell line exhibits a very high targeting efficiency (∼50%), even at the transcriptionally inactive *OVA* locus [12], it stands out as the exception in an animal kingdom wherein gene-targeting is notoriously difficult. Targeted integration is mediated by homologous recombination (HR) in both yeast and animal cells, but the reason why yeast and DT40 cells show such a high targeting ratio compared to other cells remains unknown.

HR plays a critical role in maintaining genome stability by repairing DNA double-strand breaks (DSBs)[13]. In the current model for HR-mediated DSB repair, DSBs are initially processed to produce a 3’ single-strand (ss) overhang, along which Rad51 is polymerized [14–16]. The resulting Rad51-DNA filament then searches and invades the intact homologous duplex DNA, leading to the formation of the D-loop structure. DNA synthesis from the invading strand followed by dissociation from the homologous DNA and subsequent re-annealing of the newly synthesized strand with the other end of the DSB completes the repair. This type of HR is referred as synthesis-dependent strand anneal (SDSA) and it results in sequence transfer from the intact template sequence (donor) to the damaged DNA (recipient) [15, 17]. Extensive strand exchange of the D-loop, another type of HR, leads to the generation of Holliday junction (HJ) intermediates and it results in crossovers (the exchange of strands).

In yeast and avian DT40 cells, the efficiency of targeted integration is critically reduced in mutants exhibiting attenuated HR [18, 19]. This indicates that HR is involved in targeted integration, but does not explain the underlying mechanism. A clue was discovered in the recently established gene-targeting method using designed endonucleases, such as TALEN and CRISPR. These endonucleases introduce DSBs in the targeted genome of the host chromosome, thereby promoting gene-targeting. This endonuclease-mediated targeting method allows us to utilize a targeted-gene knockout technique in various model organisms [20–24]. Successful gene-targeting by these techniques indicates that targeting integration initiates from DSBs on the host genome. The most convincing explanation for the integration mechanism is that the DSB ends on the host genome invade the homologous sequence of the plasmid and synthesize DNA along the homologous arm of the plasmid. If DNA synthesis continues past the marker gene and extends to the next arm sequence, further re-annealing with the opposite DSB end by SDSA, targeted integration should be complete. In yeast and DT40 cells, targeted integration occurs efficiently without endonuclease mediation, suggesting that these organisms have a distinct gene-targeting mechanism that remains unknown.

To explore the gene-targeting mechanism in yeast and DT40 cells, we measured the targeted-integration rate in yeast and DT40 cells using a plasmid construct digested either on the inside of the arm or on the outside of the arm. We found that DSBs formed on the homologous arm activate targeted integration, suggesting that HR is initiated by DSBs on the plasmid construct and mediates gene-targeting, presumably via crossover. Indeed, the targeted integration of gapped plasmids accompanied by crossover was efficiently induced in DT40 cells. Taking advantage of this finding, we developed a flip-in integration system for avian DT40 by utilizing the efficient nature of site-directed integration from gapped plasmids in the DT40 cells.

## Materials and Methods

### Fission Yeast Strains, Genetic Methods, and Media

General genetic procedures were carried out as described previously [25]. The standard rich yeast-extract medium, YEL (with 2% glucose), was used to culture cells. Minimal medium (SD) was used to test the uracil and leucine auxotrophy/prototrophy [26]. Transformation was performed using the lithium acetate method, as previously described [27]. The genotype of the *S*. *pombe* strain used in this study was *h* ^*+*^ *ura4-D18*.

### DT40 Culture and DNA transfection

DT40 cell line was gift from Dr. Takeda [12]. DT40 cells were cultured in an RPMI 1640 medium (Nacalai Tesque, Kyoto, Japan) supplemented with 10% heat-inactivated fetal bovine serum (FBS) (AusgeneX, QLD 4210, Australia), 1% chicken serum (GIBCO-BRL, Grand Island, NY, USA), 50 μM mercaptoethanol (Invitrogen), L-glutamin (Nacalai Tesque), 50 U/mL penicillin, and 50 μg/mL streptomycin (Nacalai Tesque). The cell lines were maintained at 39.5°C under a humidified atmosphere and 5% CO_2_. For DNA transfection, 10^7^ DT40 cells were suspended in 0.5 ml phosphate-buffered saline (PBS) containing 30μg DNA of restriction enzyme digested targeting vector and electroporated with a Gene Pulser apparatus (Bio-rad, Hercules, CA) at 550 V and 25 μF. Following electroporation, cells were transferred to 20 ml fresh medium and incubated for 24 h. Cells were then resuspended in 80 ml medium containing 0.5 μg/ml puromycinn or 25 μg/ml blastcidin depending the used maker gene, and divided into four 96-well plates. After 7–10 days, drug resistant colonies were transferred 24-well plate containing 3 ml of the fresh medium in each well and further incubated for 2 days. Genomic DNA was extracted from each expanded clone by standard procedures, and gene targeting was evaluated by Southern blot analysis or PCR.

### Construction of a *leu1* Knockout Plasmid Construct for Fission Yeast

A *leu1* gene-targeting construct was generated from a genomic sequence covering the whole leu1 ORF. A 1.2 kb *Cla*I-*Bam*HI fragment covering the *leu1* ORF was amplified with primers Fow:5’-AAAAAGGATCCTTGAGTTTCCCGAAACCAGGC-3’ and Rev:5’-AAAAAATCGATGTAATATCAGCGGTAGAAGCC-3’ and cloned in a pBlueScript SK vector. The 1.8 kb *Hind*III fragment of a *ura4* gene-expression cassette was inserted into the *Hind*III site in the *leu1* ORF to generate a *leu1* knockout construct.

### Construction of a *POLD1* Knockout Plasmid Construct for DT40

A *POLD1* knockout construct was generated from a genomic sequence including the *POLD1* gene from a genomic library. A 2.6 kb fragment of *Pst*I extending from exon 9 to exon 12 was cloned in a pBlueScript SK vector. A *HisD* selection-marker gene flanked by loxP sequences was inserted into the *Nde*I site in intron 10 to generate a *POLD1* knockout construct.

### Construction of a Flip-in System for Gene-tagging

To construct plasmids for the flip-in of the epitope tags GFP and FLAG, we modified previously described fission-yeast integration vectors int1 and int3, respectively [27]. The *LEU2* selection-gene cassette in the yeast plasmid was replaced by *Puro* and *Bsr* selection marker genes to generate int1-puro, int1-bsr, int3-puro and int3-bsr.

### Construction of a Flip-in System for the Stable Expression of *OVA* loci

To construct a plasmid for the flip-in of an expression module, a 2.8 kb *Xba*I fragment of the *OVA* gene, a fragment of the expression unit (containing β-actin promoter, multi cloning sites, and the SV40 polyA signal), and the selection marker gene (*Puro* or *Bsr*) were ligated and cloned in a pBlueScript SK vector.

### Flow-cytometric Analysis to Measure GFP-positive Cells

Flow-cytometric analysis was performed with a FACS Calibur flow cytometer (Beckton Dickinson, Mountain View, CA). Green fluorescent was detected using the FL1 channel. Dead cells were stained with propidium iodide and detected using the FL2 channel. Data were acquired and analyzed using CellQuest software.

### Measurement of Cellular Sensitivity

10^4^ cells were seeded into 24-well plates containing 1 mL/well of culture medium and incubated at 39.5°C. ATP assays were carried out with 96-well plates using a CellTiter-Glo Luminescent Cell Viability Assay Kit (Promega Corp., Madison, WI, USA) at 48 h after chemical exposure. Briefly, we transferred 100 μL cell suspensions to the individual wells of the 96-well plates, held the plates at room temperature for approximately 5 min, added 100 μL of CellTiter-Glo reagent, and mixed the contents for 5 min on an orbital shaker to induce cell lysis. The plate was then incubated at room temperature for 10 min to stabilize the luminescent signal. We measured luminescence using a Fluoroskan Ascent FL fluorometer (Thermo Fisher Scientific Inc., Waltham, MA, USA).

## Results

### The Presence of DSBs on Both Homologous Arms Stimulates Gene-Targeting in Yeast and Chicken DT40 Cells

**Fig 1:**
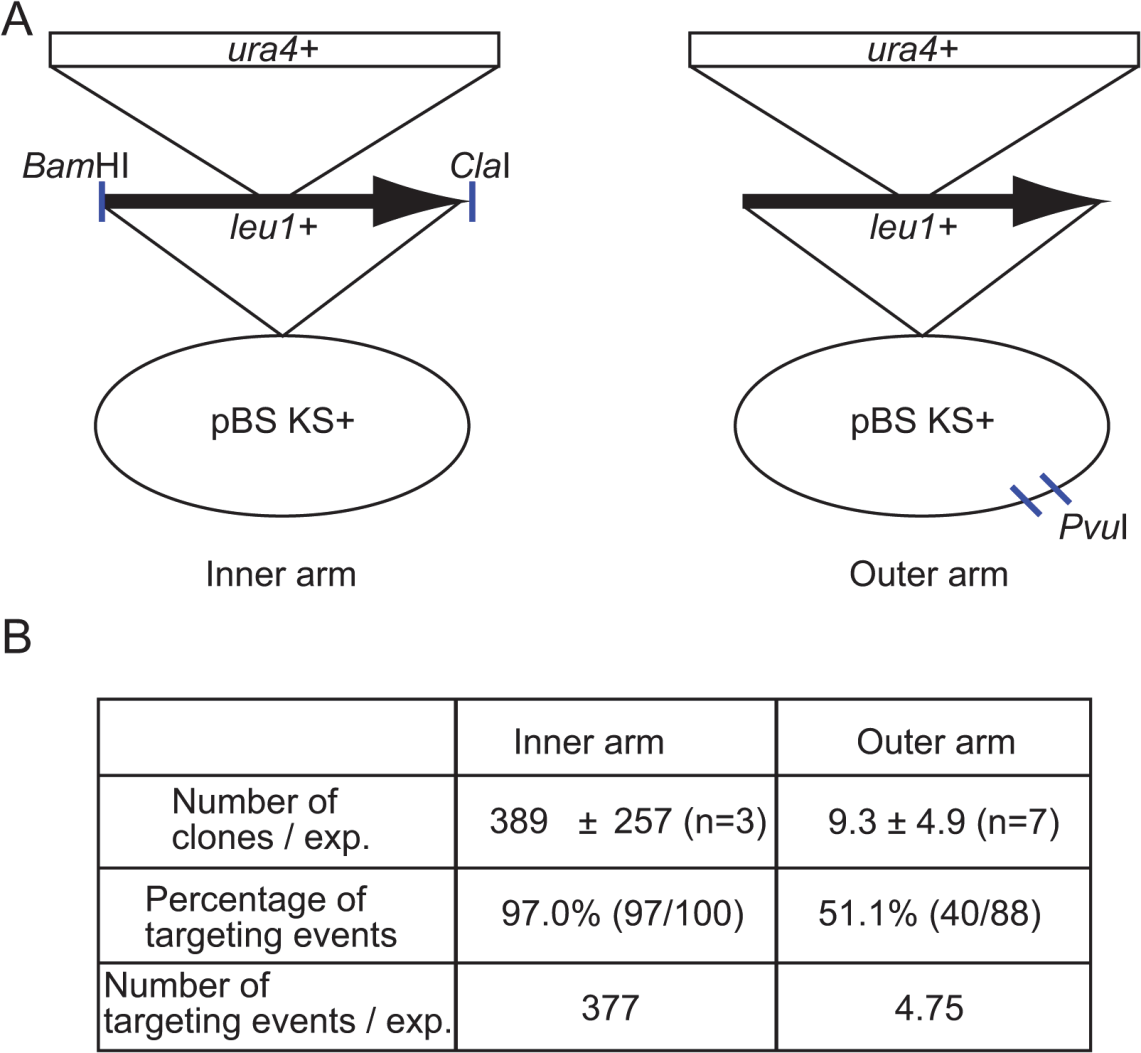
Gene-targeting at the *leu1* locus in fission yeast. (A) Schematic representation of the *leu1* knockout construct. A *Bam*HI/*Cla*I fragment containing the *leu1* ORF was cloned in a pBlueScript vector and a *ura4* marker gene was inserted in the *Hin*dIII site in the middle of the *leu1* ORF to generate a *leu1* knockout construct. The resultant knockout vector was digested at the edges of both homologous arms using *Bam*HI and *Cla*I (left), or digested in the pBlueScript vector using *Pvu*II (Right). (B) A digested knockout vector (5 μg) was transformed into a fission yeast strain (*h* ^*+*^ *ura4-D18*). The number of transformants selected for uracil prototrophy, percentage of clones showing leucine auxotrophy, and the estimated number of gene-targeting events per transformation are shown.

To understand the molecular mechanism of gene-targeting in yeast, we compared the *leu1* gene-targeting construct digested at the inner homologous arm with that digested at the outer arm (Fig. 1A). The gene-targeting construct is incapable of autonomous replication in fission yeast. Thus transformation with such a plasmid is much less efficient than with replicable plasmids, since they must integrate into the host chromosome. Targeted integration on the *leu1* gene was measured by leucine auxotrophy during cell growth. Transformation efficiency was over 40 times higher with the inner arm digested plasmid than with the outer arm one (Fig. 1B). Moreover, the targeting rate was higher in the clones transformed with the construct digested at the inner arm. Construct digested at the inner arm generated about 80 times more recombinant than did the outer arm digested one in fission yeast (Fig. 1B).

**Fig 2:**
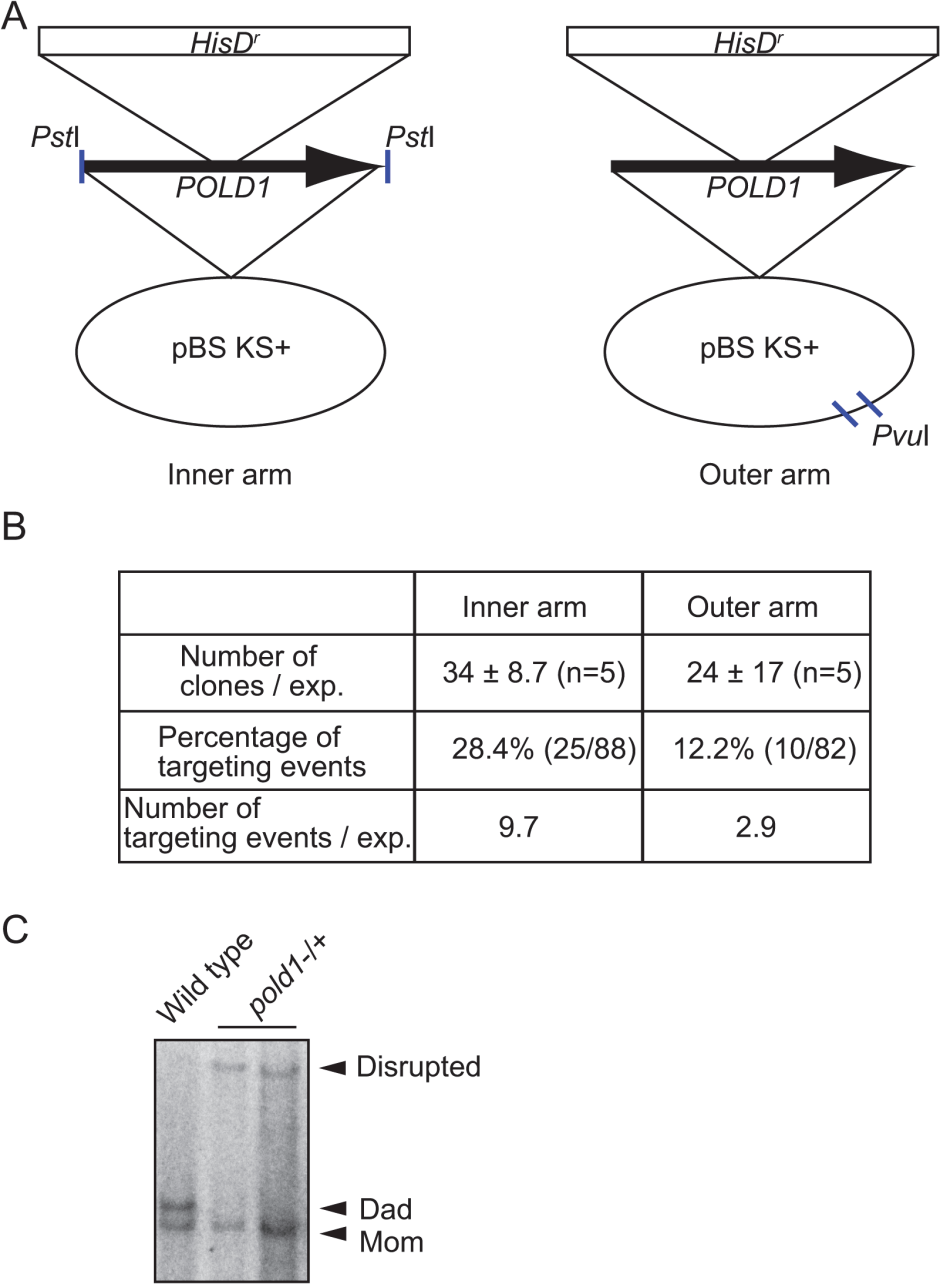
Gene-targeting at the *POLD1* locus in DT40 cells. (A) Schematic representation of the *POLD1* knockout construct. A *Pst*I fragment containing a part of the *POLD1* locus was cloned in a pBlueScript vector and a *hisD*-resistant marker gene was inserted in the *Nde*I site in the middle of the *POLD1* fragment to generate a *POLD1* knockout construct. The resultant knockout vector was digested at the edges of both homologous arms using *Pst*I (left), or digested in the pBlueScript vector using *Pvu*II (Right). (B) The digested knockout vector (50 μg) was transfected into DT40 cells (#653 *wild-type* cl-18). The number of colonies selected for resistance to L-histidinol, percentage of colonies in which the *POLD1* gene was disrupted, and the estimated number of gene-targeting events per transfection are shown. (C) Representative image showing Southern blot for assessing *pold1* gene disruption. Due to significant difference of the *pold1* gene locus (around exon 10) between dad and mom allele in DT40, wild type cells exhibited two bands.

We next analyzed what effect the digestion site of construct on gene targeting in DT40 cells. To this end, we compared *POLD1* gene-targeting efficiency using targeting construct digested at the inner arm and at the outer arm (Fig. 2A). *POLD1* gene-targeting was examined by Southern blot analysis. The efficiency of colony formation was slightly higher in construct digested at the inner arm than at the outer arm (Fig. 2B). Moreover, the targeting rate was significantly higher in the clones derived from construct digested at the inner arm (p = 0.016). The total enhancement of targeted integration by the inner arm digestion was over 3 times higher in DT40 cells (Fig. 2B, 2C). These data indicate that targeted integration is stimulated by construct digestion at the inner homologous arm in both fission yeast and DT40 cells. Moreover, it appears that gene-targeting is initiated from the edges of the targeting construct in these cells. Given that gene-targeting is stimulated via DSB formation on the host chromosome in mammalian cells, initiation from the edges of the targeting construct in yeast and in DT40 cells suggests that these organisms have a unique integration mechanism. This mechanism probably involves an interaction between each end of the targeting fragment and the corresponding region of homology in the chromosome. In addition, integration of the targeting fragment must occur by some mechanism other than simple SDSA accompanied by crossover. In DT40 cells, the contribution, if any, of this hypothetical mechanism to gene-targeting might be limited, because the effect of inner-arm digestion in DT40 cells is critically smaller than that in yeast cells.

### Active Homologous Integration Accompanied by Crossover in DT40 Cells

**Fig 3:**
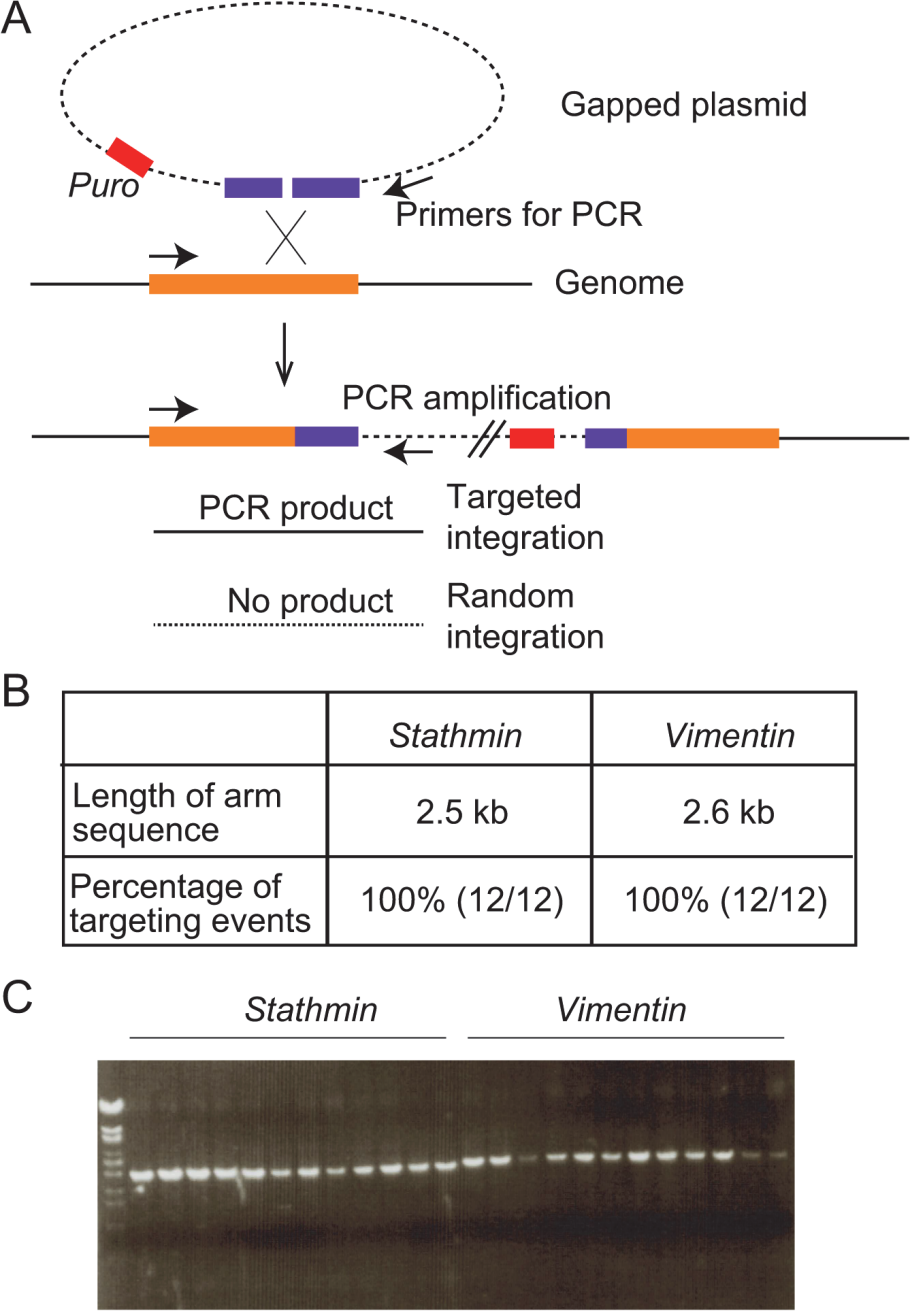
Efficient crossover-mediated homologous integration in DT40 cells. (A) Schematic representation of homologous integration using gapped plasmids. An homologous-arm sequence (purple bar) was cloned in a vector carrying a puromycin-resistant gene (red bar). The resulting vector was digested with a restriction enzyme to make the gap in the homologous sequence. Gapped plasmids were transfected into DT40 cells and homologous integration accompanied by chromosomal crossover resulted in the integration of whole plasmids into the homologous region. Targeted integration was assessed by PCR using primers (arrows). (B) Length of the homologous arm and percentage of the homologous targeting for integration in *stathmin* and *vimentin* loci. (C) Representative image showing PCR products using the primers shown in A. λ-phage DNA digested with *Eco*T14I was used as a size maker.

To discover whether or not the hypothetical integration mechanism accompanied by crossover is active in DT40 cells, we tested the integration of gapped plasmids, which has been shown to be accompanied by crossover in budding yeast [28]. We tested *stathmin* and *vimentin* gene loci for targeted integration in DT40 cells. We cloned 2.5 and 2.6 kb fragments corresponding to the 3’ region of *stathmin* and *vimentin* gene in a pBS-Puro vector carrying puromycin-resistant gene respectively. The resultant stathmin-puro and vimentin-puro vectors were digested with *BglI*I and *Nsi*I, respectively, which digest cloned homologous arms. *Bgl*II divides the 2.5 kb *stathmin* locus into 1.0 and 1.5 kb fragments. *Nsi*I divides the 2.6 kb *vimentin* locus into 0.6 and 2.0 kb fragments. The resultant gapped plasmids were transfected into DT40 cells and selected for resistance to puromycin (Fig. 3A). This gapped plasmid system generates crossover-mediated homologous integration [28]. When the rate of targeted integration was assessed by PCR (Fig. 3A) we found that all examined DNA-integration events were targeted integration (Fig. 3B, 3C). This result indicates that gene-targeting associated with crossover is critically active in DT40 cells, as in budding yeast.

### Development of a Flip-in System to Fuse Epitope Tags to Proteins of Interest

**Fig 4:**
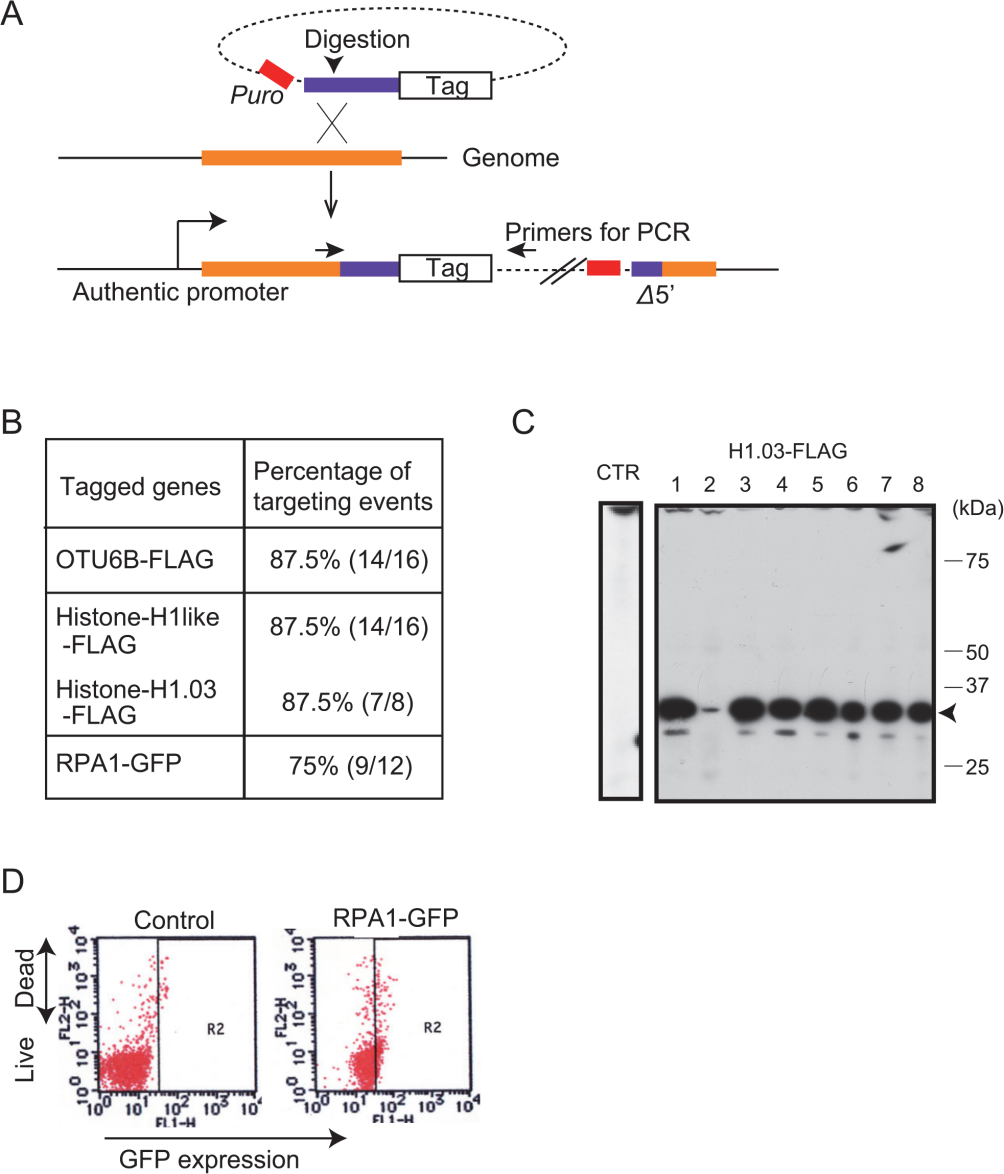
Flip-in system to fuse epitope tags to proteins of interest. (A) Schematic representation of the flip-in system to fuse epitope tags to proteins of interest. Genomic sequence containing the last exon but lacking the stop codon (purple bar) was cloned and connected with an in-frame sequence tag. The resulting targeting vector was digested in the middle of the cloned homologous arm (purple bar). Gapped plasmids were transfected. At least for 10 days the transfected cells were cultured before assessing homologous integration. Homologous integration resulted in the replacement of the original gene by the modified gene encoding the tagged protein. (B) The percentage of homologous integration for tagging OTU6B, histone H1-like protein, histone H1.03, and RPA was summarized. (C) Representative image of western blot with anti-FLAG antibody showing FLAG-tagged histone H1.03. (D) Dot-plot representation of flow-cytometric analysis to evaluate fluorescent emissions (green) from RPA-GFP. The cell was stained with propidium iodide (red) to exclude the dead-cell fraction. Strength of green and red fluorescence was plotted on the x-axis and y-axis, respectively, in a logarithmic scale.

Having established active crossover-mediated homologous integration in DT40 cells, we next applied this technique to generate a rapid flip-in system to fuse epitope tags to proteins of interest. In yeast, the targeted integration of whole plasmids into chromosomes accompanied by the modification of the host genome has been used for truncating genes, gene mutation, and gene-tagging [27, 29]. We modified plasmids [27] that were previously used in fission yeast by replacing the selection marker gene. In this system, the selection-marker gene, the 3’ region of the gene of interest, and the epitope-tag sequence are ligated (Fig. 4A). We cloned 4.6, 3.1, 2.7, and 2.3 kb fragments corresponding to the 3’ region of genes encoding OTU6B, histone H1-like protein, histone H1.03, and RPA lacking stop codon, respectively and fused them in-frame to the epitope tag. Each plasmid was then digested with a restriction enzyme that cuts one site in the cloned gene, resulting in gapped plasmids with homologous arms at their edges. When the rate of targeted integration was assessed by PCR (Fig. 4A, 4B) we found that the efficiency of targeted integration was over 70% in all constructs examined. FLAG-tagged OTU6B, histone H1-like protein, and histone H1.03 were detected by western blot analysis (Fig. 4C and data not shown). GFP-tagged RPA was also detected using flow-cytometric analysis (Fig. 4D). These data indicate that this flip-in system is useful for replacing endogenous genes with modified genes such as a tag-fusion gene. The advantage of this system is that modified genes can be expressed at physiological expression levels.

### Development of a Flip-in System to Stably Express Genes of Interest

**Fig 5:**
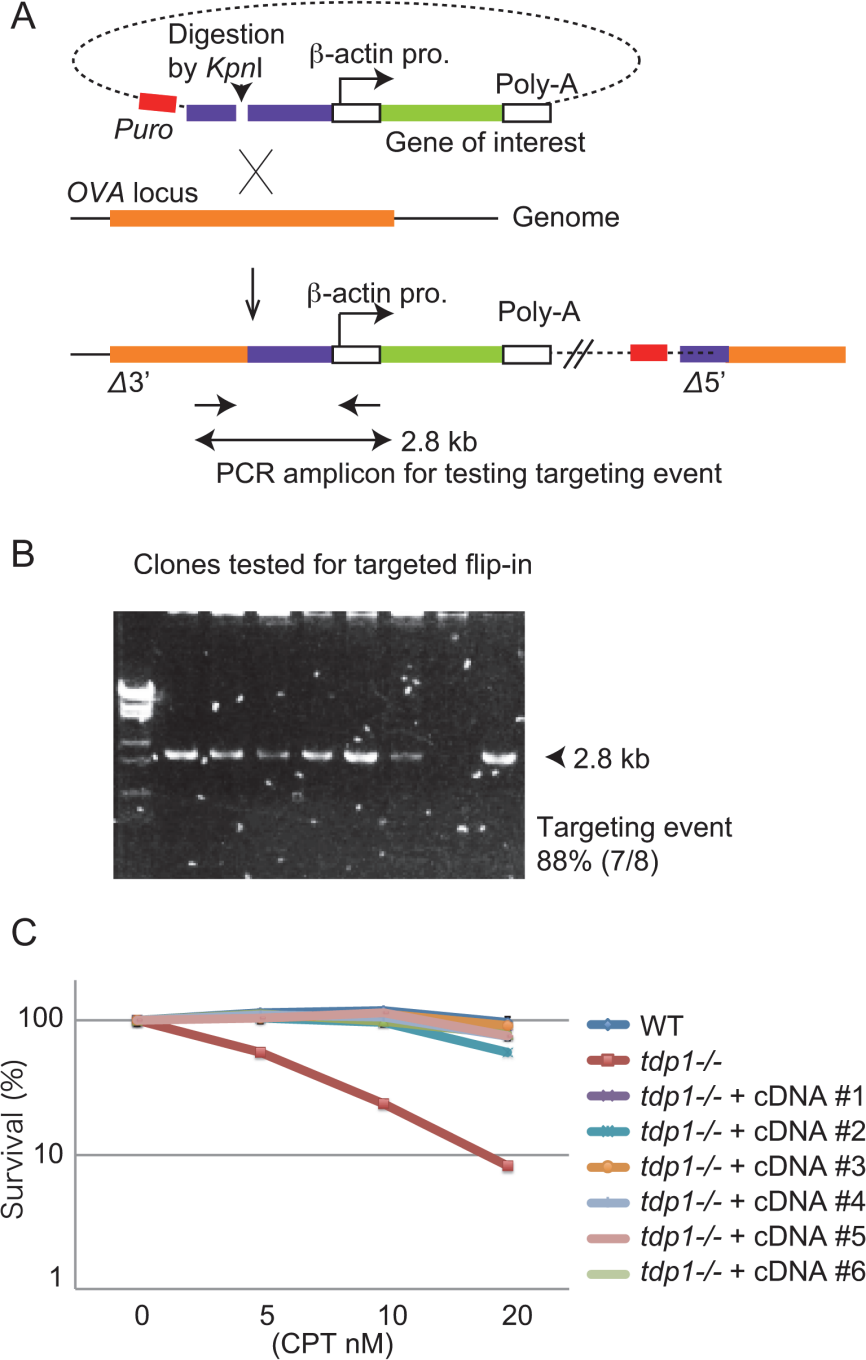
Flip-in system to stably express transgenes of interest. (A) Schematic representation of a flip-in system to stably express transgenes. A genomic sequence containing a 2.8 kb fragment of an *OVA* gene (purple bar) was cloned and connected with a puromycin-resistant gene and an expression cassette (β-actin promoter, multi-cloning site and polyadenylation signal). The resulting targeting vector was digested with *Kpn*I and transfected. At least for 10 days the transfected cells were cultured before assessing homologous integration. Homologous integration results in the integration of the expression cassette into the *OVA* locus. Target integration was assessed by PCR using primers (arrows). (B) Representative image showing PCR products using the primers shown in A. λ-phage DNA digested with *Eco*T14I was used as a size maker. (C) Complementation of camptothecin hypersensitivity of *tdp1* ^-/-^ cells by stable expression of human *TDP1* cDNA. Cellular sensitivity to camptothecin was analyzed. Survival rate was calculated as the percentage of surviving cells treated with DNA-damaging agents relative to untreated surviving cells. Error bars show the SD of mean for three independent assays. The concentration is displayed on the horizontal axis, while the survival rate is displayed on the y-axis on a logarithmic scale.

We next developed a gene-expression module using a flip-in system. We chose the *OVA* locus in chromosome 2 to integrate the expression cassette, since the *OVA* locus is transcriptionally inactive and known to exhibit a high targeting rate in DT40 cells. We placed the selection marker gene, a 2.7 kb *Xba*I fragment of the *OVA* locus, and an expression cassette consisting of β-actin promoter, cloning sites, and poly-A sites into a plasmid (Fig. 5A). To activate HR, we digested the plasmid with *Kpn*I to cut one site in the *OVA* fragment, resulting in gapped plasmids with homologous arms at their edges. When the rate of targeted integration was assessed by PCR (Fig. 5A, 5B), we found that the efficiency of targeted integration was over 80%. Using this system, we examined the genetic complementation of null phenotypes by expression of cloned cDNA. We cloned human *TDP1* cDNA encoding the enzyme Tyrosyl-DNA phosphodiesterase 1, which is required for cellular tolerance to camptothecin [30, 31]. We tested *tdp1* ^-/-^ cells and six *tdp1* ^-/-^ clones complemented with *human-TDP1* cDNA for cellular tolerance to camptothecin. The *tdp1* ^-/-^ cells exhibited a greater sensitivity than did *wild-type* cells [30, 32], while the sensitivity of the *tdp1* ^-/-^ clones was nearly the same as that of the *wild-type* cells. This result suggests that our method of stable cDNA expression has an advantage over the conventional method for stable expression via random integration. This is because the expression levels of random integrant clones should vary according to the locus of the integration.

## Discussion

In this study, we explored the mechanism underlying efficient gene-targeting in yeast and DT40 cells. We found that targeting constructs containing DSBs at both ends of the homologous arms enhance targeting efficiency by 80 times in fission yeast cells and by 3 times in DT40 cells. This suggests a hypothetical gene-targeting mechanism in yeast and DT40 cells wherein gene-targeting initiates from DSBs at both edges of the homologous arms in the targeting construct.

This is in marked contrast to gene-targeting in mammalian cells, because it initiates from DSBs introduced into homologous chromosomal DNA. This is evidenced by the fact that gene-targeting in mammalian cells can be activated by DSB formation on the homologous region of a chromosome [20–24]. In yeast and DT40 cells, each end of the targeting fragment might interact with the corresponding region of homology in the chromosome, and the integration of the targeting fragment must occur by some mechanism other than simple SDSA accompanied by crossover. The contribution of this hypothetical mechanism to gene-targeting might be limited in DT40 cells, however, because the magnitude of gene-targeting enhancement caused by the presence of DSBs on both ends of the homologous arms in the DT40 cells was only one twenty fifth of that in the fission yeast cells.

The gene-targeting mechanism in budding yeast has been analyzed with great precision. It has been suggested that gene-targeting is initiated by two independent strand invasions resulting in chromosomal crossover [33]. In DT40 cells, it is also likely that the end of the targeting fragment might interact with the corresponding chromosome region, leading to recombination associated with crossover. Indeed, we detected efficient homologous integration accompanied by crossover in DT40 cells (Fig. 3). Such crossover-mediated targeted integration is also reported as occurring in mouse ES cells, but the efficiency is critically low [34]. Considering that most gene-targeting initiates from DSBs on the homologous region of a chromosome and is probably mediated with simple SDSA in animal cells, the magnitude of the contribution of crossover-mediated targeted integration in gene-targeting might be different across species and cell type.

We developed flip-in systems for the rapid generation of cells expressing tag-fused proteins and the stable expression of transgenes from the *OVA* locus in DT40 cells by taking advantage of the efficient nature of DT40 cells in terms of gene-targeting via crossover-mediated integration. In the first system, endogenous genes can be replaced by FLAG- or GFP-tagged fusion genes. The resulting tag-fused genes expressed from an authentic promoter had an expression level that might reach nearly physiological levels. To avoid experimental artifacts caused by over-expression, our system may provide a novel opportunity for analyzing genes of interest at physiological levels of expression. Note that genes whose expression is under the regulation of 3’ UTR (untranslated region) might be altered in this flip-in system due to the change of 3’UTR sequence. The second system involves the stable expression of genes at a defined level of expression. In this system, an expression module consisting of a β-actin promoter, genes of interest, and a polyadenylation signal can be targeted in *OVA* loci. In the standard system, the expression modules might be randomly integrated in the chromosomes, thus the expression level can be critically different from clone to clone. Such differences make precise comparisons of phenotypes in random integration clones impossible. Our system allows us to express transgenes from defined locus, thereby enabling a more precise comparison of clones. Moreover, our system provides high long-term stability of the transgene expression, since we detected transgene expression at least 10 days (corresponding to 30 times cell divisions) after transfection of the plasmid (Fig. 5, data not shown). Furthermore, we detected constant expression over one month (data not shown).

In conclusion, we have suggested a hypothetical gene-targeting pathway in avian DT40 cells and have developed a rapid flip-in system that takes advantage of the unique nature of avian DT40 cells.
